# Extensive weight loss reveals distinct gene expression changes in human subcutaneous and visceral adipose tissue

**DOI:** 10.1038/srep14841

**Published:** 2015-10-05

**Authors:** Adil Mardinoglu, John T. Heiker, Daniel Gärtner, Elias Björnson, Michael R. Schön, Gesine Flehmig, Nora Klöting, Knut Krohn, Mathias Fasshauer, Michael Stumvoll, Jens Nielsen, Matthias Blüher

**Affiliations:** 1 Department of Biology and Biological Engineering, Chalmers University of Technology, SE-412 96, Gothenburg, Sweden; 2 Science for Life Laboratory, KTH - Royal Institute of Technology, SE-171 21, Stockholm, Sweden; 3 University of Leipzig, Department of Medicine, Leipzig, Germany; 4 Städtisches Klinikum Karlsruhe, Clinic of Visceral Surgery, Karlsruhe, Germany; 5 IFB Adiposity Diseases, Junior Research Group 2 “Animal models of obesity”; 6 Core Unit DNA-Technologies, Interdisziplinäres Zentrum für Klinische Forschung (IZKF) Leipzig, Germany

## Abstract

Weight loss has been shown to significantly improve Adipose tissue (AT) function, however changes in AT gene expression profiles particularly in visceral AT (VAT) have not been systematically studied. Here, we tested the hypothesis that extensive weight loss in response to bariatric surgery (BS) causes AT gene expression changes, which may affect energy and lipid metabolism, inflammation and secretory function of AT. We assessed gene expression changes by whole genome expression chips in AT samples obtained from six morbidly obese individuals, who underwent a two step BS strategy with sleeve gastrectomy as initial and a Roux-en-Y gastric bypass as second step surgery after 12 ± 2 months. Global gene expression differences in VAT and subcutaneous (S)AT were analyzed through the use of genome-scale metabolic model (GEM) for adipocytes. Significantly altered gene expressions were PCR-validated in 16 individuals, which also underwent a two-step surgery intervention. We found increased expression of cell death-inducing DFFA-like effector a (CIDEA), involved in formation of lipid droplets in both fat depots in response to significant weight loss. We observed that expression of the genes associated with metabolic reactions involved in NAD+, glutathione and branched chain amino acid metabolism are significantly increased in AT depots after surgery-induced weight loss.

Obesity is a complex disorder with various contributing genetic, psychological and environmental factors and it is characterized by deposition of an excess amount of fat in the adipose tissue (AT). Obesity has become one of the most serious public health problems of the 21^st^ century and its worldwide prevalence continues to rise not only in adults but also in children and adolescents. Globally, nearly 1.5 billion adults and 43 million preschool children are overweight (body mass index (BMI) > 25 kg/m^2^) or obese (BMI > 30 kg/m^2^)^1^,^2^. Epidemiologic studies have shown that obesity is associated with increased mortality and that it is a major risk factor in the progression of serious medical conditions including type 2 diabetes (T2D), cardiovascular disease, non-alcoholic fatty liver disease (NAFLD) and certain cancers^3^.

Fat deposition in visceral depots makes obese individuals more prone to complications than subcutaneous fat accumulation^4^. Adipocytes and other AT cells from subcutaneous (S) and visceral (V) ATs release a plethora of bioactive molecules, metabolites, lipids, proteins and hormones (adipokines)^5^. Adipokines mediate the crosstalk of AT with major organs and tissues, and thus modulate whole-body metabolism, inflammatory and immune processes^6^. Even though a multidisciplinary research effort involving clinical, genetic, biochemical, pharmacological and omics approaches is engaged, the underlying molecular mechanisms for the progression of obesity associated diseases in adipocytes have not been fully elucidated. On that note, the mechanisms underlying beneficial effects of significant weight loss on obesity related diseases and associated risk factors also remain unclear.

In multiple studies, weight loss has been shown to improve disease risk factors and prevent T2D development in obese individuals^7^,^8^,^9^. However, significant weight loss is only achieved by intensive life-style intervention and maintaining long-term weight loss or at least preventing weight regain remain major challenges^10^,^11^. Furthermore, life-style interventions have not been proven to be effective in the long term clinical treatment of morbid obesity^12^. Previous retrospective cohort studies have shown that bariatric surgery (BS) is the most effective intervention to reduce body weight, obesity associated disease risk factors and overall mortality in morbidly obese individuals in the long term^13^,^14^,^15^. The use of BS has increased dramatically during the past decade due to an acceptable risk/benefit profile^16^. Considerable metabolic improvements are seen in morbidly obese individuals after BS and further mechanistic insights into the activated mechanisms in response to BS will potentially lead to non-invasive treatments for obesity^17^,^18^.

There are already cross-sectional data available for global gene expression profiling in SAT and VAT of obese individuals^19^,^20^,^21^,^22^,^23^,^24^,^25^. Moreover, proteins in adipocytes have been characterized and used for the reconstruction of a GEnome-scale metabolic Model (GEM) for adipocytes, *iAdipocytes1809*^26^. GEMs are the collection of the biochemical reactions and associated enzymes in a cell/tissue type and they provide an excellent scaffold for analyzing the complexity of metabolism^26^,^27^,^28^,^29^,^30^,^31^. These integrative models are employed for prediction of complex phenotypes from genotypes in health and disease states^27^,^32^,^33^,^34^,^35^,^36^,^37^,^38^,^39^,^40^,^41^,^42^,^43^. Recently, the content of *iAdipocytes1809* has been validated through the use SAT RNA sequencing data and updated based on the latest proteomics data in Human Protein Atlas (www.proteinatlas.org)^44^,^45^,^46^,^47^,^48^,^49^ to reconstruct *iAdipocytes1850*^50^. In this context, GEM for adipocytes has been used for the integration of omics data of healthy and obese individuals and for the analysis of the SAT transcriptomics data of lean and obese individuals involved in the Swedish Obese Subjects Sib Pair study^26^,^50^.

Here, we performed two-step BS with sleeve gastrectomy as first and a Roux-en-Y gastric bypass surgery as second step in 22 severely obese individuals (Table 1). In order to increase our knowledge about the complexity of biological processes associated with BS, we obtained SAT and VAT samples from the same individuals at the two surgery time points, and performed global gene expression profile analyses of SAT and VAT for six of the severely obese individuals using microarrays. Using these data, we revealed the biological processes altered in SAT and VAT of the obese individuals in response to BS through integrative analysis engaging *iAdipocytes1850.* We tested in an unbiased microarray approach the hypotheses that extensive weight loss affects the gene expression profiling of VAT and SAT, and causes alterations in pathways associated with AT energy and lipid metabolism, inflammation, and secretory function (e.g. adipokines). Finally, we validated a subset of our significant findings by quantitative reverse transcription PCR (RT-PCR) methods using SAT and VAT samples obtained from 16 independent individuals that underwent BS.

## Results

### Characteristics of study participants

In 22 severely obese individuals, we performed a two step bariatric surgery strategy (first step: sleeve gastrectomy; second step after 12 ± 2 months: Roux-en-Y gastric bypass surgery). Anthropometric and circulating parameters of the participants are shown in Table 1 and Supplementary Dataset 1 before and after the extensive weight loss. BS caused an average 42 kg of body weight and 14.5 BMI reduction in obese individuals involved in our study (Table 1). We collected SAT and VAT samples as previously described^51^ from these 22 individuals before and after weight loss, and generated microarray gene expression data for a subgroup of six representative individuals. The independent SAT and VAT samples from 16 individuals were used to validate significantly altered gene expression by RT-PCR. To identify a potential gender bias, we tested gene expression changes after significant weight loss validated by QRT-PCR separately for men and women and confirmed all gene expression changes independently of the gender. Moreover, we did not find any differences in the expression of the validated genes between men and women.

Several clinical variables significantly changed and indicated improved health status in these severely obese individuals (Table 1). Parameters related to insulin resistance and T2D including fasting plasma glucose (FPG), fasting plasma insulin (FPI) and hemoglobin A1c (HbA1c) were all significantly decreased indicating improved insulin sensitivity after weight loss. HDL cholesterol and circulating adiponectin significantly increased and fasting plasma triacylglycerol (TG) was significantly decreased and indicated improved blood lipid status. Hypertriglyceridemia was evident (fasting TG > 1.7 mmol/L) in 11 out of 22 individual before the weight loss but only in one individual after the weight loss. C-reactive protein (CrP), leptin serum concentrations and liver function parameters alanine aminotransferase (ALAT), aspartate aminotransferase (ASAT) and γ-Glutamyl transferase (gGT) were significantly decreased in response to weight loss (Table 1). Interestingly, LDL cholesterol and total cholesterol were both within the normal range at baseline and did not change during the study. Overall, the likely increase in insulin sensitivity, the large reduction in fasting triglycerides and the increase in HDL cholesterol strongly indicated improved health status of the individuals in response to weight loss.

### Global gene expression profiling of SAT and VAT

**Figure 1:**
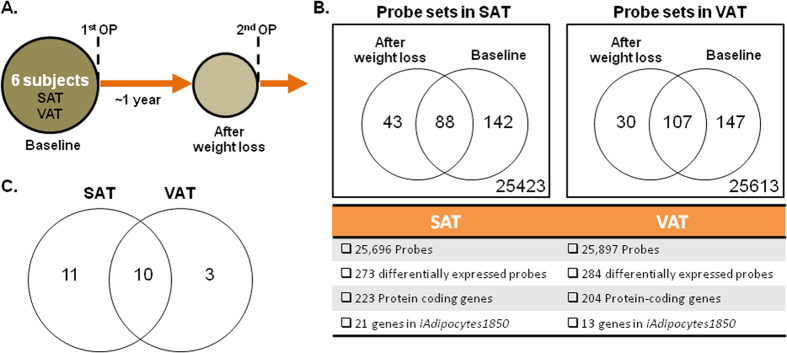
Global gene expression profiling of VAT and SAT before and after extensive weight loss. (**A**) VAT and SAT samples were obtained from individuals who underwent two-step bariatric surgery and microarray data were generated. (**B**) Gene expression data for different adipose tissue depots was analyzed independently by comparing to the gene expression data of lean individuals. Differentially expressed probe sets, protein coding genes as well as the metabolic genes in *iAdipocytes1850* were identified. (**C**) The overlap between the differentially expressed metabolic genes in *iAdipocytes1850* in two different adipose tissue depots were shown and used in network dependent analysis.

Gene expression profiling of AT depots was performed using Illumina HumanHT-12 V4.0 whole genome expression chip for six of the individuals involved in our study (Fig. 1A). Due to the heterogeneity of the BMI before (40.9 < BMI < 65 kg/m^2^) and after (26.7 < BMI < 54.6 kg/m^2^) extensive weight lost, we compared the expression level of genes in AT depots of obese individuals to healthy lean individuals (internal additional control) for hereby, identifying significantly differentially expressed genes before and after the extensive weight loss, and finally detected the direction of the changes in the expression of the genes (Fig. 1B). Differential expression analysis was carried using the Piano R package^52^ and false discovery rate (FDR) adjusted p-values (Q-values) were calculated. In total, 273 probe sets in the microarray chip were significantly (Q-values < 0.05) differentially expressed in SAT samples and 284 probe sets were significantly (Q-values < 0.05) differentially expressed in VAT samples comparing to the reference individuals. The complete list of the genes whose expression that changed in SAT and VAT after extensive weight loss is presented in Supplementary Dataset 2.

We carried out gene set analysis for Gene Ontology (GO) biological process (BP) terms to obtain a general perspective on the changes in SAT and VAT likely to result from the weight loss and calculated the gene set statistics using reporter algorithm implemented in the Piano R package^52^. We performed GO analysis by comparing global SAT and VAT gene expression of individuals before and after weight loss to the gene expressions of lean individuals and presented the significantly enriched GO BP terms for SAT and VAT before and after the weight loss (Figure S1–S4). We observed that GO BP terms associated with immune response, regulation of protein secretion and lipid metabolism particularly lipid storage, negative regulation of lipid catabolic process and low-density lipoprotein particle clearance were significantly enriched after the extensive weight loss.

In SAT, expression of genes including integrin, beta 2 (ITGB2), lipopolysaccharide binding protein (LBP), lymphocyte cytosolic protein 1 (LCP1), matrix metallopeptidase 9 (MMP9), secreted phosphoprotein 1 (SPP1) and Thy-1 cell surface antigen (THY1) associated with the GO Term “immune system process” were significantly decreased after extensive weight loss. Moreover, in VAT, expression of aquaporin 9 (AQP9), complement factor B (CFB), interferon regulatory factor 1 (IRF1), proteoglycan 4 (PRG4) and metalloproteinase inhibitor 1 (TIMP1) associated with the GO Term “immune system process” were significantly decreased after extensive weight loss. This indicated the decreased immune response of the AT depots after weight loss.

### Differences in SAT and VAT lipid metabolism in response to BS

**Figure 2:**
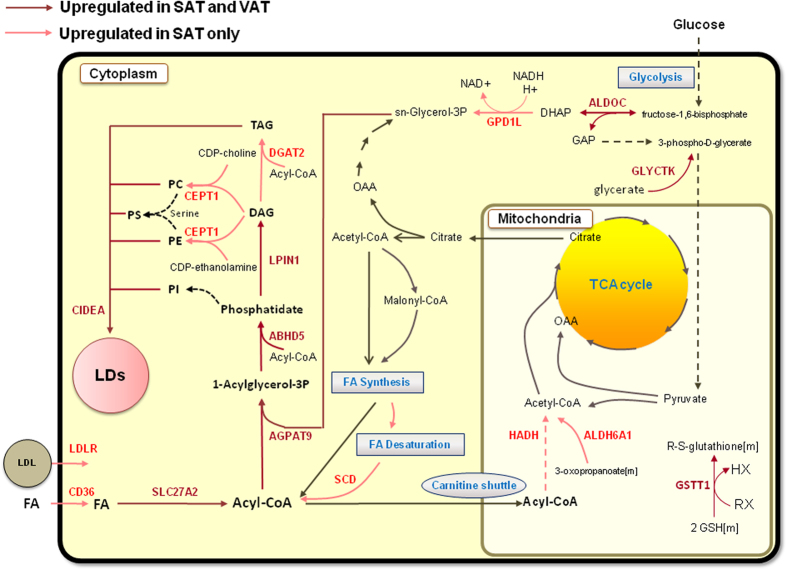
Increased formation of lipid droplet in VAT and SAT of obese individuals after extensive weight loss. Even though limited number of metabolic genes was differentially expressed in both adipose tissue depots, the expression of several genes involved in the formation of lipid droplets was increased. Red and dark red arrows indicated the significant upregulation of the associated genes in SAT only (light red) and in both SAT and VAT (dark red), respectively.

**Figure 3:**
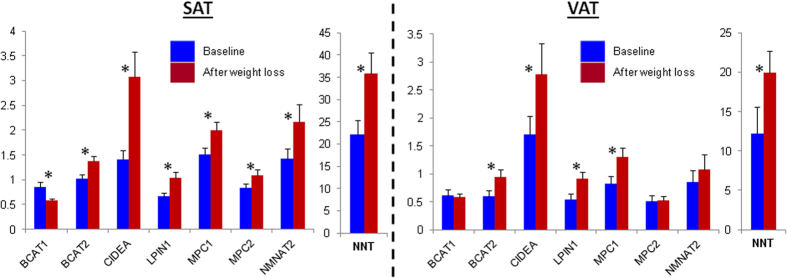
Validation of the gene expression changes before and after extensive weight loss. The expression levels of CIDEA and LPIN1 involved in the formation of lipid droplets, BCAT1 and BCAT2 involved in the catabolism of the branch chain amino acids, NMNAT2 involved in the NAD^+^ salvage pathway, NNT involved in the glutathione synthesis and MCP1 and MCP2 involved in the transport of pyruvate to the mitochondria before and after extensive weight loss were calculated by quantitative RT-PCR. Each bar represents the results from before and after extensive weight loss for 16 SAT and VAT samples, and mean ± standard error of the mean (SEM) values are presented. *Student’s t test was used and P-value < 0.05 was considered statistically significant.

In order to get a general overview about the expression changes of metabolic genes (genes in *iAdipocytes1850*) in SAT and VAT in response to weight loss, detailed metabolic differences were identified by mapping the significantly differentially expressed genes on the network topology provided by *iAdipocytes1850* (Figure S5). We identified lipid metabolism particularly the formation of lipid droplet (LD) in both SAT and VAT as most strongly affected metabolic pathway in response to extensive weight loss (Fig. 2). Through network analysis, we found that expression of cell death-inducing DFFA-like effector a (CIDEA) as well as lipin 1 (LPIN1), abhydrolase domain containing 5 (ABHD5), 1-acylglycerol-3-phosphate O-acyltransferase 9 (AGPAT9), aldolase C, fructose-bisphosphate (ALDOC) involved in the formation of LDs were increased in SAT and VAT of obese individuals after weight loss. Moreover, we found that the expression of diacylglycerol O-acyltransferase 2 (DGAT2), choline/ethanolamine phosphotransferase 1 (CEPT1), CD36 molecule (CD36), low density lipoprotein receptor (LDLR) and stearoyl-CoA desaturase 5 (SCD) involved in the lipid metabolism are increased in SAT after weight loss (Supplementary Dataset 2). We analyzed the expression levels of CIDEA and LPIN1 in the SAT and VAT samples obtained from an independent cohort of 16 obese individuals by quantitative RT-PCR and found that expression of these two genes were significantly (Student’s t test, P-value < 0.05) increased after BS (Fig. 3).

Moreover, we found that the expression of lipolytic genes including hormone-sensitive lipase, LIPE (HSL) and patatin-like phospholipase domain-containing protein 2, PNPLA2 (ATGL) were significantly altered by extensive weight loss (p < 0.05). However, after multiple testing the calculated Q-value was higher than 0.05 which we used as a cut of for detecting the statistically significantly changed genes in our study.

### Increased expression of the genes catalyzing glutathione and NAD+ related reactions

We observed that the expression of glutathione s-transferase theta 1 (GSTT1) gene associated with reactions that use glutathione as cofactor is increased in both SAT and VAT after weight loss (Fig. 2). Glutathione can be synthesized from amino acids glutamate, cysteine and glycine through *de novo* glutathione synthesis pathways. It is a key metabolite for the reduction of the damage to cellular components caused by reactive oxygen species (ROS), such as H_2_O_2_. In order to perform this vital function, cells need to maintain a pool of glutathione in the reduced state (GSH) and this requires an increased pool of glutathione through *de novo* biosynthesis.

Weight loss may lead to a significant increase in glutathione levels since genes catalyzing reactions that use glutathione as substrate were significantly (Q-values < 0.05) upregulated. We first checked the expression of enzymes involved in glutathione synthesis by RT-QPCR and observed that none of these enzymes (GCLC, GCLM and GSS) were significantly different in SAT and VAT after weight loss. Glutathione may also be regenerated from its oxidized form, GSSG by glutathione reductase (GR) that requires NADPH as a substrate. The resulting NADPH is directly linked to GSH synthesis. Hence, we measured the expression of Nicotinamide nucleotide transhydrogenase (NNT) that catalyzes mitochondrial transmembrane hydride transfer between NADH and NADP+ to generate NADPH in SAT and VAT of the individuals before and after the weight loss by RT-QPCR. We found that expression of the NNT is significantly (Student’s t test, P-value  < 0.05) increased in the SAT and VAT after extensive weight loss (Fig. 3). Taken together, these observations indicate that individuals who underwent BS may have higher total glutathione levels and these could be explained by the increased expression of NNT in AT depots.

In addition, expression of nicotinamide nucleotide adenylyltransferase 2, NMNAT2 involved in the NAD+ salvage pathways in SAT as well as changes in the expression NAD+ consuming enzymes in SAT and VAT increased after weight loss (Figure S5). Intracellular levels of NAD+ play a major role in maintaining the regular metabolic status of the cell and the total NAD+ pool within the cell is stabilized between *de novo* and salvage biosynthetic pathways and its utilization. It has been earlier reported that the main sources of NAD+ within the cell are the salvage pathways^53^ and these require uptake of NAD+ precursors including nicotinamide D-ribonucleotide and nicotinamide ribonucleoside from outside of the cells. We confirmed increased expression of NMNAT2 in SAT by RT-PCR (Fig. 3). On the other hand, we could not detect any significant changes in the expression of NMNAT2 in VAT of obese individuals after weight loss based on microarray and RT-PCR data.

Previously, it has been shown that AT is the major source of the elevated plasma ROS in obese mice when other tissues including the liver, skeletal muscle, and aorta are examined^54^. We observed that the expression of genes (NMNAT2) involved in the synthesis of NAD+ was increased selectively in SAT after extensive weight loss.

### Increased expression of genes associated with the mitochondrial energy metabolism

Mitochondrial dysfunction due to impaired oxidative phosphorylation is a well-established consequence of obesity. Intracellular levels of NAD+ play a key role in the homeostatic control of mitochondrial function of the cell. We hypothesized that increased levels of NAD+ after extensive weight loss could also boost the mitochondrial activity in both fat depots of obese individuals.

**Figure 4:**
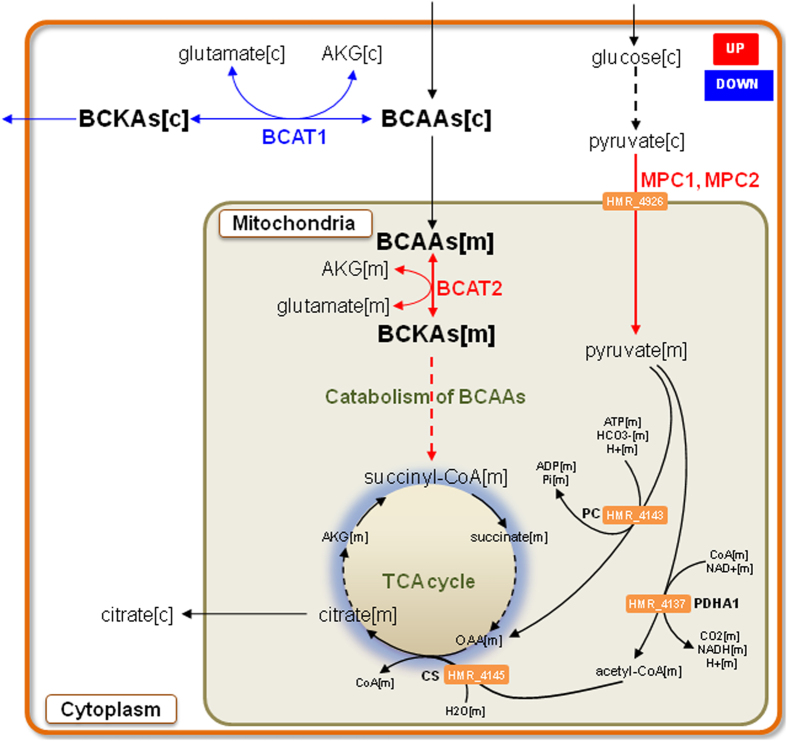
Increased expression of the genes associated with the mitochondrial metabolic activity in SAT and VAT after extensive weight loss. Model for the expression of the genes involved in the transport of pyruvate from cytosol to mitochondria as well as the expression of the genes involved in the catabolism of branch chain amino acids in mitochondria is increased after extensive weight loss. Changes in gene expression before and after the extensive weight loss are highlighted in either red (higher expression) or blue (lower expression).

Previously, we have reported that the expression of the mitochondrial pyruvate carrier 1 (MPC1) and 2 (MPC2) that are essential for several major pathways of carbohydrate, fat, and amino acid metabolism^55^ are decreased in obesity^50^. In order to confirm the increased expression of the genes in the mitochondria after weight loss, we analyzed the expression of MPC1 and MPC2 using quantitative RT-PCR. We found that the expression of the MPC1 and MPC2 were significantly increased in SAT (Student’s t test, P-value  < 0.05) whereas the expression of MPC1 was significantly increased (Student’s t test, P-value < 0.05) in VAT after weight loss (Fig. 3). Increased expression of MPC1 and MPC2 in obese individuals after weight loss may indicate the increased mitochondrial activity in SAT and VAT of obese individuals having weight loss (Fig. 4). Moreover, we found that the expression of phosphoenolpyruvate carboxykinase 2 (PCK2), hydroxyacyl-CoA dehydrogenase (HADH) and aldehyde dehydrogenase 6 family, member A1 (ALDH6A1) are increased in the SAT mitochondria after weight loss.

### Alterations in the branched-chain amino acids metabolism

The branched-chain amino acids (BCAAs), leucine, isoleucine, and valine, are essential amino acids and it has been earlier reported that levels of BCAAs are elevated in plasma of obese individuals^56^. We found that expression of the ALDH6A1 involved in the catabolism of the BCAA is significantly increased in SAT after BS. We have previously shown that plasma levels of BCAAs are increased due to decreased catabolism of BCAAs in SAT of obese individuals^50^. The catabolism of BCAA starts with transamination of BCAAs to α-keto acids (BCKAs) by cytosolic and mitochondrial branched-chain aminotransferases, BCAT1 and BCAT2, respectively. BCKAs can be released into the circulation, and liver can decarboxylate BCKAs to acyl coenzyme A (acyl-CoA) derivatives by the rate-limiting enzyme, branched-chain a-ketoacid dehydrogenase (BCKDH). We have previously reported that expression of BCAT1 is increased whereas the expression of BCAT2 is decreased in the SAT of obese individuals compared to lean individuals^50^.

We hypothesized that increased mitochondrial activity of both AT depots after BS, may result in increased catabolism of BCAAs. Hence, we compared the expression of BCAT2 in SAT and VAT of obese individuals before and after weight loss by quantitative RT-PCR analysis (Fig. 3) and found that expression of BCAT2 is increased in both SAT and VAT after BS (Fig. 4). Moreover, we found that the expression of BCAT1 is decreased in SAT after weight loss. On the other hand we could not detect significant changes in the expression of BCAT1 in VAT of obese individuals after extensive weight loss.

## Discussion

AT stores and mobilizes triglycerides and has a major role in regulating whole-body metabolism. Using whole genome expression arrays, we determined SAT and VAT global gene expression changes in six obese individuals undergoing a two-step BS strategy causing extensive weight loss after sleeve gastrectomy (first step surgery). Gene expression profiling of both SAT and VAT in the same individual after significant weight loss allowed us to delineate biological processes most likely related to weight loss. We observed that significant weight loss is associated with significant changes in blood pressure, TGs, HDL-cholesterol, CrP, adiponectin, FPG and FPI. These multiple significant changes in glucose and lipid metabolism as well as AT function in response to weight loss are significant confounding factors, which may either in a concerted mode or as single factors confound the observed gene expression changes. Our study design does therefore not allow drawing conclusions with regard to the specific causative factors of gene expression changes.

LDs are an important subcellular organelle that are found in adipocytes and have fundamental roles in metabolism. LDs protect the cell against lipotoxicity by sequestering excess fat and have roles in the development of obesity, T2D and NAFLD. We found that expressions of the SAT enriched gene CIDEA as well as other genes involved in the formation of LDs are increased after extensive weight loss. CIDEA is highly enriched in human SAT compared to other major 32 human tissues in healthy individuals^50^ and it regulates the TG deposition in AT. It has been reported that the expression of CIDEA in VAT of obese individuals correlates positively with whole-body insulin sensitivity similar to the known LD protein perilipin^57^. LPIN1 is also highly expressed in human SAT^50^ and its expression positively correlated with insulin sensitivity whereas it inversely correlated with parameters of adiposity^58^. We have previously found that the expression of the CIDEA and LPIN1 is significantly downregulated in the SAT of male and female obese individuals compared to lean individuals^50^ and the expression of both genes were increased after extensive weight loss in morbidly obese subjects.

Cidea inhibits the brown adipose tissue uncoupling process in rodents and Cidea-null mice are lean and resistant to diet-induced obesity^59^. The association between CIDEA and basal metabolic have also been elucidated in human obesity and it has been found that its expression is inversely associated with basal metabolic rate and uncoupling protein 1 (UCP1) expression^60^. Decreased expression of CIDEA has been linked to high body fat content and high insulin levels in obese subjects. In addition to its role in lipid metabolism, Cidea has been found as a novel target gene for both peroxisome proliferator-activated receptor (PPAR)α and -γ in the mouse liver and the induction of Cidea in liver with PPARα and -γ agonists has suggested a possible role for Cidea in energy metabolism^61^.

The expression of CIDEA was also increased in cancer cachexia, a condition associated with extensive weight loss^62^. It has also been shown that the expression of BCAT2 changes in response to cachexia^63^ and we found that expression of BCAT2 also changed in SAT and VAT of the individuals after extensive weight loss. There is a direct link to cachexia via CIDEA, as studies suggest that basal lipolysis is further decreased in human fat cells in cancer cachexia and this might be due to enhanced AT levels of CIDEA which decrease basal lipolysis in human AT. Cancer cachexia patients suffer from serious weight loss, primarily AT mass. Cachexia associated gene expression changes in AT mainly involve an upregulation of energy turnover regulating pathways, whereas genes controlling intra- and extracellular architecture were downregulated^63^. Similar changes have been found as a result of weight-loss in obese individuals^64^,^65^. Obesity, opposite to cachexia is associated with a state of chronic inflammation, but inflammatory genes were not found affected by SAT gene expression changes observed in cachexia^66^. In contrast, weight-loss is associated with significant downregulation of inflammatory pathways in the AT stromal vascular fraction^67^. Based on these findings, one might speculate that rapid and significant weight loss after BS results in changes in AT gene expression profile towards a cachexia-associated pattern accompanied by improved metabolic and disease risk factors via additional changes in inflammatory gene expression.

Alterations in immune pathways were found in obese individuals and the level of inflammation was significantly decreased after extensive weight loss. We found that the expression of genes associated with immune processes were significantly decreased in both SAT and VAT. On the other hand, we detected significant differences in the plasma level of adiponectin and leptin, before and after weight loss but could not detected any significant changes in the expression of these genes in SAT and VAT of the individuals before and after weight loss.

The expected changes in the expression of lipolytic genes after the extensive weight loss were also detected in our analysis. However, after multiple testing the calculated Q-value was higher than 0.05 which is used as a cut of for detecting the statistically significantly changed genes in our study. One plausible explanation can be the small sample size used in our study. In addition, we cannot exclude that a body weight maintenance phase two months prior to the 2^nd^ step Roux-en-Y surgery may have caused the absence of expected gene expression changes in lipolytic genes. Another limitation of our study is that due to the relatively small sample size, a paired sample analysis could not be performed. Therefore further studies are required to confirm our results using larger cohorts and/or different methods (e.g. RNA sequencing) for the quantification of gene expression changes after significant weight loss.

In order to address obesity in a wider population of affected individuals, less invasive treatments than BS are needed. Our results can be used for understanding the underlying molecular mechanisms of the clinical and physiological effects of BS and designing less invasive and potentially safer interventions to mimic the effects of BS. In summary, we identified previously unrecognized gene expression changes in AT in response to extensive weight loss, which may represent targets for the future development of pharmacological weight loss treatments.

## Materials and Methods

### Study-subject characteristics

From 22 Caucasian obese volunteers (17 females and 5 males), plasma samples, SAT and VAT biopsies were obtained in the context of a two-step bariatric surgery strategy with gastric sleeve resection as the first step and a Roux-en-Y- gastric bypass as second step surgery 12 ± 2 months after sleeve gastrectomy. The baseline BMI in this subgroup was 53.1 ± 10.3 kg/m^2^ and the BMI after gastric sleeve resection was 38.6 ± 6.8 kg/m^2^ (Table 1).

SAT and VAT samples obtained from six individuals (the first six women who completed the 2^nd^ step surgery and in which AT RNA quality (tested by Agilent RNA 6000 Nano Kit, Agilent Technologies, Santa Clara, CA, USA) was sufficient for microarray experiments) at both surgery time points were used for microarray experiments and additional SAT and VAT samples obtained from 16 individuals (recruited subsequently after the first 6 individuals) were used to validate gene expression changes detected by microarray by RT-QPCR (Table 1). All study participants had a stable weight with no fluctuations of more than 2% of the body weight for at least three months before surgery. Individuals with severe conditions including generalized inflammation or end stage malignant diseases were excluded from the study. VAT samples were obtained from the upper left part of the omentum. VAT and SAT samples were immediately frozen in liquid nitrogen after explanation. The study was approved by the Ethics Committee of the University of Leipzig (Reg. number: 017-12-23012012). The methods were carried out in accordance with approved guidelines and regulations. All participants gave written informed consent before taking part in the study.

### Global transcriptome profiling of SAT and VAT

Total RNA was extracted as previously described^68^ and SAT and VAT gene expressions were measured using Illumina HumanHT-12 V4.0 whole genome expression chip (Illumina Inc, San Diego, USA). Pre-processing of the raw data was done using Illumina software, GenomeStudio (Illumina Inc., San Diego). Chip background effects were corrected and the average bead signal and detection p-values were calculated using negative controls present on the array. Original raw data includes expression levels with chip background levels subtracted and detection p-values for over 47,000 probes.

The output of the GenomeStudio was uploaded to the MATLAB and the quantilenorm function was used to apply quantile normalization. SAT and VAT samples processed separately and probes which did not have detection p-value < 0.05 in any of the samples were removed. The “genelowvalfilter” function was used to filter out genes with very low absolute expression and “genevarfilter” function to filter out genes with a small variance across samples were applied to the normalized data. 25,696 and 25,897 probes were retained for SAT and VAT samples respectively.

Due to the difference between the size of the adipocytes in the SAT and VAT samples, the microarray data were in addition normalized to AT depot specific reference genes. The use of the reference genes depends on the tissue type and the experimental conditions. Throughout our analysis, the β-glucuronidase (GUSB) gene and β-Actin (ACTB) were used as reference in SAT and VAT, respectively. GUSB was reported as the best reference gene in SAT due to its steady mRNA level at different body weights and fat mass across the replicate samples^69^. Similarly, ACTB was suggested as a reference gene in the gene expression studies of the human VAT samples^70^.

We compared gene expression profiles in SAT and VAT of severe obese individuals with that of lean individuals. In parallel, a Singular Value Decomposition (SVD) and a standard clustering analysis were employed to identify gross patterns of the transcriptome. P-values were corrected for multiple testing and analysis of variance (ANOVA) was used to determine significance of differences between ratios at Q-values < 0.05 and log2 fold changes > 0.1.

### RT-PCR

SAT and VAT samples were immediately frozen in liquid nitrogen after explanation. Total RNA was extracted from frozen SAT and VAT obtained from 16 obese male and female individuals underwent BS before and after the surgery using RNeasy Lipid Tissue Mini Kit (Qiagen, Hilden, Germany). Assay-on-demand (ABI, Foster City, USA) probes were used for the quantitative RT-PCR, performed in a 96-well LightCycler 480 system. Expression levels of CIDEA, LPIN1, NMNAT2, MPC1, MPC2, NNT, BCAT1 and BCAT2 were calculated relative to the mRNA expression of GUSB in SAT and ACTB in VAT and calculated with the ΔΔ-Ct method.

## Additional Information

**How to cite this article**: Mardinoglu, A. *et al.* Extensive weight loss reveals distinct gene expression changes in human subcutaneous and visceral adipose tissue. *Sci. Rep.* **5**, 14841; doi: 10.1038/srep14841 (2015).

## Supplementary Material

Supplementary Information

Supplementary Dataset 1

Supplementary Dataset 2

## Figures and Tables

**Table 1:** Clinical characteristic of the study participants.

Characteristic	Baseline	12 months post-surgery	P-value
Body weight (kg)	152 ± 36.3	110 ± 21.1	5.6E-05
BMI (kg/m^2^)	53.1 ± 10.1	38.6 ± 6.69	3.2E-06
Fasting plasma glucose (FPG) (mmol/l)	6.87 ± 1.81	5.16 ± 0.71	4.0E-04
Fasting plasma insulin (FPI) (pmol/l)	191 ± 145	66.5 ± 46.1	9.6E-04
HbA1c (%)	6.23 ± 0.93	5.31 ± 0.50	3.6E-04
Triacylglycerol (TG) (mmol/l)	1.78 ± 0.56	1.10 ± 0.36	4.2E-05
Free fatty acids (FFA) (mmol/l)	0.93 ± 0.25	0.37 ± 0.20	5.1E-10
Total cholesterol (mmol/l)	4.97 ± 0.93	4.73 ± 0.74	0.34
LDL-cholesterol (mmol/l)	3.14 ± 0.67	2.97 ± 0.84	0.51
HDL-cholesterol (mmol/l)	1.04 ± 0.31	1.39 ± 0.28	4.8E-04
C-reactive protein (CrP) (mg/l)	6.11 ± 3.26	2.26 ± 1.39	2.9E-05
Alanine aminotransferase (μkat/l)	0.83 ± 0.41	0.48 ± 0.25	1.8E-03
Aspartate transaminase (μkat/l)	0.66 ± 0.33	0.39 ± 0.16	2.3E-03
γ-Glutamyl transferase (μkat/l)	0.82 ± 0.63	0.41 ± 0.18	8.5E-03
Leptin (ng/ml)	57.9 ± 16.9	33.0 ± 10.6	1.8E-06
Adiponectin (μg/ml)	4.88 ± 3.04	10.3 ± 2.25	9.1E-08

Data are shown as means ± SD. P-value indicates significance-level of difference before and after weight loss for the 22 individuals undergoing bariatric surgery.
