# Supplementary material for: Extensive weight loss reveals distinct gene expression changes in human subcutaneous and visceral adipose tissue
Source: Sci Rep. 2015 Oct 5;5:14841. doi: 10.1038/srep14841 (PMC4593186; doi:10.1038/srep14841)

Extensive weight loss reveals distinct gene expression changes in human subcutaneous and visceral adipose tissue

Running title: **Adipose tissue changes after bariatric surgery**

Adil Mardinoglu[1,2,*], John T. Heiker[3], Daniel Gärtner[4], Elias Bjornson[1], Michael R. Schön[4], Gesine Flehmig[3], Nora Klöting[5], Knut Krohn[6], Mathias Fasshauer[3], Michael Stumvoll[3], Jens Nielsen[1,2] and Matthias Blüher[3,*]

[1]Department of Biology and Biological Engineering, Chalmers University of Technology, SE-412 96, Gothenburg, Sweden.

[2]Science for Life Laboratory, KTH - Royal Institute of Technology, SE-171 21, Stockholm, Sweden.

[3]University of Leipzig, Department of Medicine, Leipzig, Germany.

[4]Städtisches Klinikum Karlsruhe, Clinic of Visceral Surgery, Karlsruhe, Germany.

[5]IFB Adiposity Diseases, Junior Research Group 2 "Animal models of obesity".

[6]Core Unit DNA-Technologies, Interdisziplinäres Zentrum für Klinische Forschung (IZKF) Leipzig, Germany.

*Corresponding authors

E-mail: matthias.blueher@medizin.uni-leipzig.de
Tel: +49 341 9713829
Fax: +49 341 9722439

E-mail: adilm@chalmers.se
Tel: +46 31 772 3140
Fax: +46 31 772 3801

SUPPLEMENTARY FIGURE LEGENDS

Figure S1 The enrichment of differentially expressed genes in SAT of obese subjects before the bariatric surgery compared to lean subjects was identified using Gene Ontology Biological Process (GO BP) terms. The most significantly enriched GO BP terms are presented.

Figure S2 The enrichment of differentially expressed genes in SAT of obese subjects after the bariatric surgery compared to lean subjects was identified using Gene Ontology Biological Process (GO BP) terms. The most significantly enriched GO BP terms are presented.

Figure S3 The enrichment of differentially expressed genes in VAT of obese subjects before the bariatric surgery compared to lean subjects was identified using Gene Ontology Biological Process (GO BP) terms. The most significantly enriched GO BP terms are presented.

Figure S4 The enrichment of differentially expressed genes in VAT of obese subjects after the bariatric surgery compared to lean subjects was identified using Gene Ontology Biological Process (GO BP) terms. The most significantly enriched GO BP terms are presented.

Figure S5 A cellular integrative model of the most significant changes in the expression of metabolic genes in A) VAT and B) SAT of obese subjects after extensive weight loss is presented. Red and blue arrows and indicates the upregulation and downregulation of the associated reactions, respectively. Names of genes with higher expression after significant weight loss are in red, genes with lower expression in blue.

SUPPLEMENTARY DATASETS LEGENDS

Supplementary Dataset 1 Clinical characteristic of each subjects involved in our study before the sleeve gastrectomy and ~1 year post-surgery (before and after extensive weight loss).

Supplementary Dataset 2 List of the differentially expressed probe sets in SAT and VAT identified by comparing the expression of the genes before and after the extensive weight loss to the expression of genes in lean subjects.

Figure S1

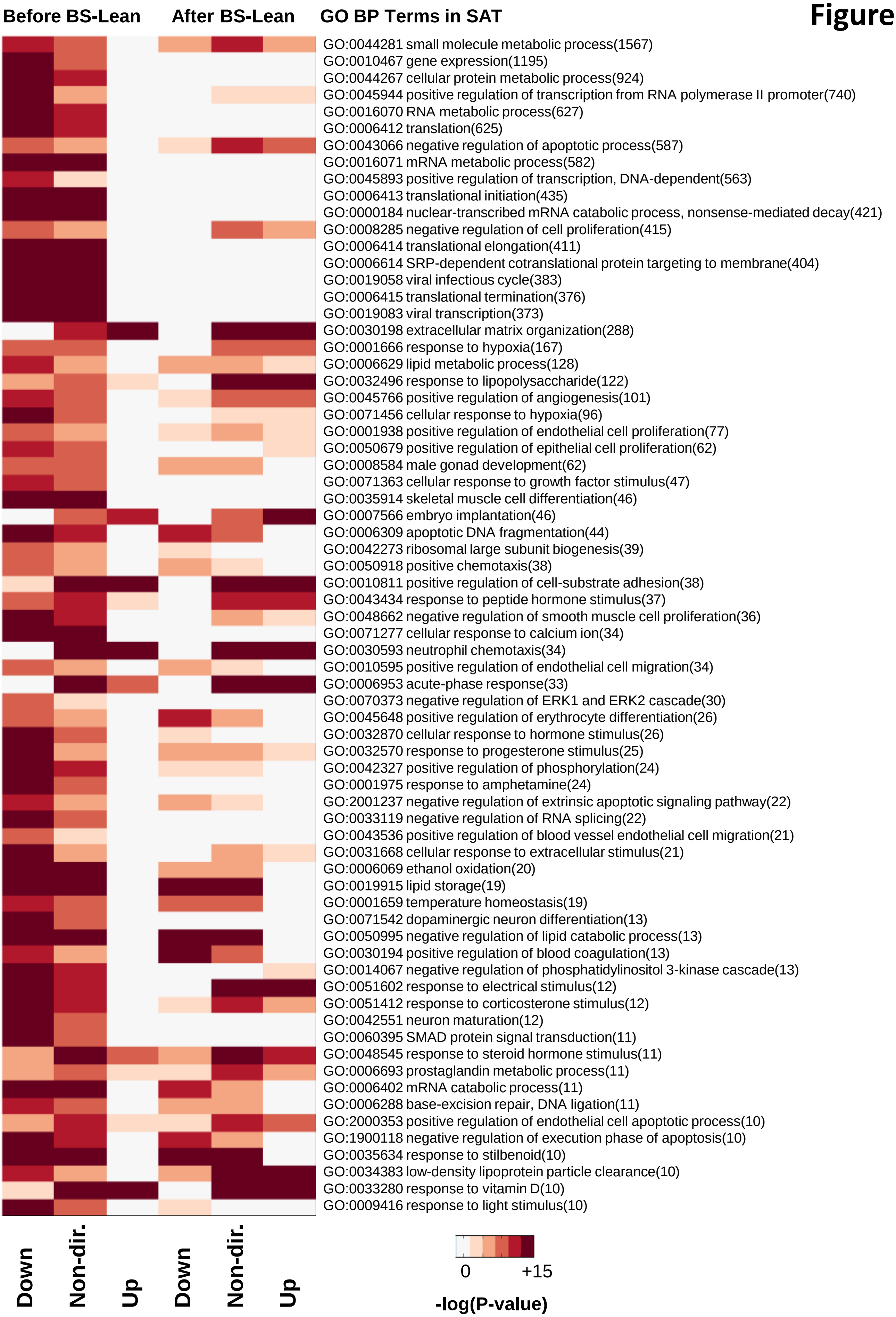
Before BS-Lean
After BS-Lean
GO BP Terms in SAT
GO:0044281 small molecule metabolic process(1567)
GO:0010467 gene expression(1195)
GO:0044267 cellular protein metabolic process(924)
GO:0045944 positive regulation of transcription from RNA polymerase II promoter(740)
GO:0016070 RNA metabolic process(627)
GO:0006412 translation(625)
GO:0043066 negative regulation of apoptotic process(587)
GO:0016071 mRNA metabolic process(582)
GO:0045893 positive regulation of transcription, DNA-dependent(563)
GO:0006413 translational initiation(435)
GO:0000184 nuclear-transcribed mRNA catabolic process, nonsense-mediated decay(421)
GO:0008285 negative regulation of cell proliferation(415)
GO:0006414 translational elongation(411)
GO:0006614 SRP-dependent cotranslational protein targeting to membrane(404)
GO:0019058 viral infectious cycle(383)
GO:0006415 translational termination(376)
GO:0019083 viral transcription(373)
GO:0030198 extracellular matrix organization(288)
GO:0001666 response to hypoxia(167)
GO:0006629 lipid metabolic process(128)
GO:0032496 response to lipopolysaccharide(122)
GO:0045766 positive regulation of angiogenesis(101)
GO:0071456 cellular response to hypoxia(96)
GO:0001938 positive regulation of endothelial cell proliferation(77)
GO:0050679 positive regulation of epithelial cell proliferation(62)
GO:0008584 male gonad development(62)
GO:0071363 cellular response to growth factor stimulus(47)
GO:0035914 skeletal muscle cell differentiation(46)
GO:0007566 embryo implantation(46)
GO:0006309 apoptotic DNA fragmentation(44)
GO:0042273 ribosomal large subunit biogenesis(39)
GO:0050918 positive chemotaxis(38)
GO:0010811 positive regulation of cell-substrate adhesion(38)
GO:0043434 response to peptide hormone stimulus(37)
GO:0048662 negative regulation of smooth muscle cell proliferation(36)
GO:0071277 cellular response to calcium ion(34)
GO:0030593 neutrophil chemotaxis(34)
GO:0010595 positive regulation of endothelial cell migration(34)
GO:0006953 acute-phase response(33)
GO:0070373 negative regulation of ERK1 and ERK2 cascade(30)
GO:0045648 positive regulation of erythrocyte differentiation(26)
GO:0032870 cellular response to hormone stimulus(26)
GO:0032570 response to progesterone stimulus(25)
GO:0042327 positive regulation of phosphorylation(24)
GO:0001975 response to amphetamine(24)
GO:2001237 negative regulation of extrinsic apoptotic signaling pathway(22)
GO:0033119 negative regulation of RNA splicing(22)
GO:0043536 positive regulation of blood vessel endothelial cell migration(21)
GO:0031668 cellular response to extracellular stimulus(21)
GO:0006069 ethanol oxidation(20)
GO:0019915 lipid storage(19)
GO:0001659 temperature homeostasis(19)
GO:0071542 dopaminergic neuron differentiation(13)
GO:0050995 negative regulation of lipid catabolic process(13)
GO:0030194 positive regulation of blood coagulation(13)
GO:0014067 negative regulation of phosphatidylinositol 3-kinase cascade(13)
GO:0051602 response to electrical stimulus(12)
GO:0051412 response to corticosterone stimulus(12)
GO:0042551 neuron maturation(12)
GO:0060395 SMAD protein signal transduction(11)
GO:0048545 response to steroid hormone stimulus(11)
GO:0006693 prostaglandin metabolic process(11)
GO:0006402 mRNA catabolic process(11)
GO:0006288 base-excision repair, DNA ligation(11)
GO:2000353 positive regulation of endothelial cell apoptotic process(10)
GO:1900118 negative regulation of execution phase of apoptosis(10)
GO:0035634 response to stilbenoid(10)
GO:0034383 low-density lipoprotein particle clearance(10)
GO:0033280 response to vitamin D(10)
GO:0009416 response to light stimulus(10)
Down
Non-dir.
Up
Down
Non-dir.
Up
0
+15
-log(P-value)

Figure S2

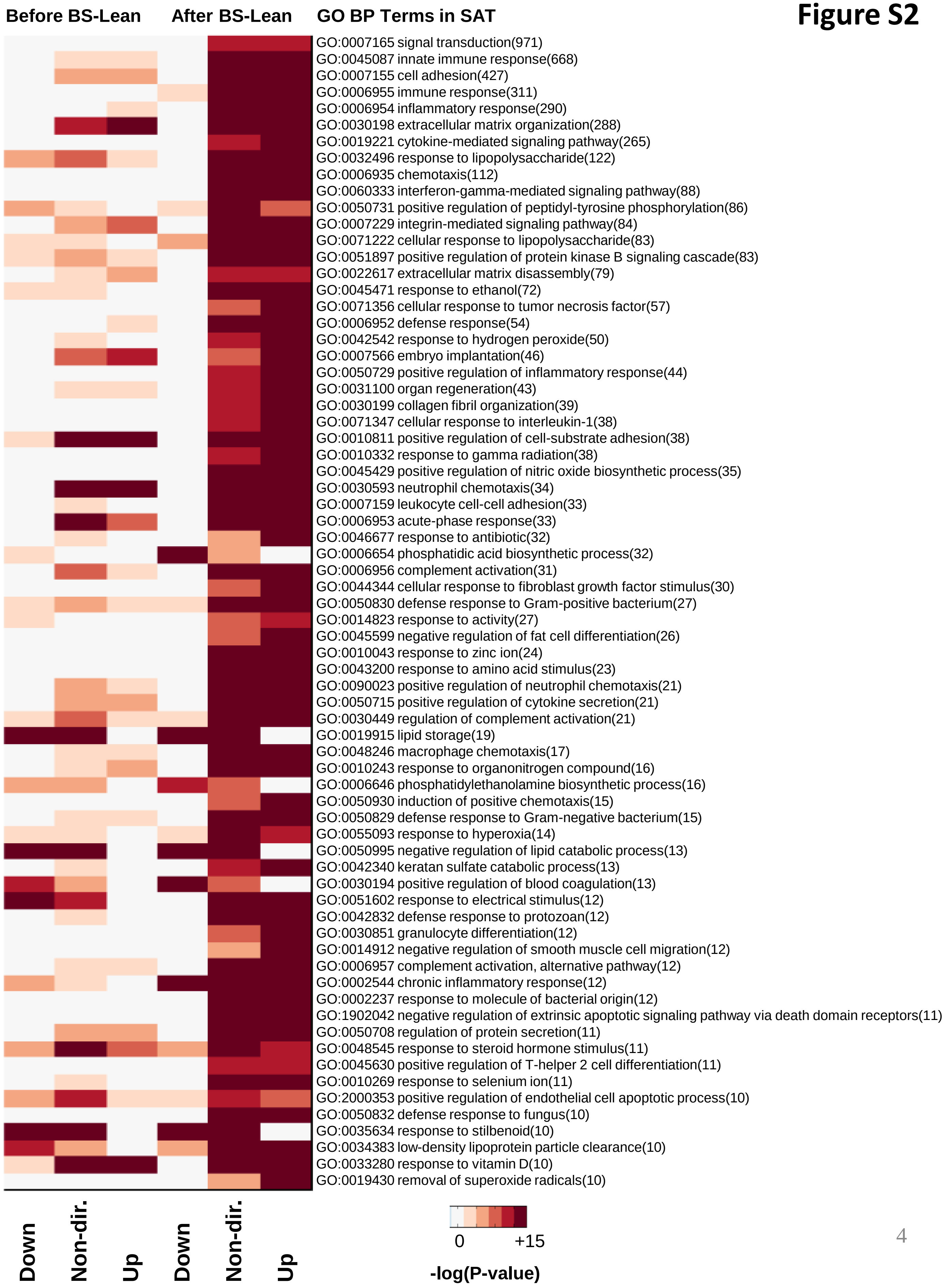

Before BS-Lean
After BS-Lean
GO BP Terms in SAT
GO:0007165 signal transduction(971)
GO:0045087 innate immune response(668)
GO:0007155 cell adhesion(427)
GO:0006955 immune response(311)
GO:0006954 inflammatory response(290)
GO:0030198 extracellular matrix organization(288)
GO:0019221 cytokine-mediated signaling pathway(265)
GO:0032496 response to lipopolysaccharide(122)
GO:0006935 chemotaxis(112)
GO:0060333 interferon-gamma-mediated signaling pathway(88)
GO:0050731 positive regulation of peptidyl-tyrosine phosphorylation(86)
GO:0007229 integrin-mediated signaling pathway(84)
GO:0071222 cellular response to lipopolysaccharide(83)
GO:0051897 positive regulation of protein kinase B signaling cascade(83)
GO:0022617 extracellular matrix disassembly(79)
GO:0045471 response to ethanol(72)
GO:0071356 cellular response to tumor necrosis factor(57)
GO:0006952 defense response(54)
GO:0042542 response to hydrogen peroxide(50)
GO:0007566 embryo implantation(46)
GO:0050729 positive regulation of inflammatory response(44)
GO:0031100 organ regeneration(43)
GO:0030199 collagen fibril organization(39)
GO:0071347 cellular response to interleukin-1(38)
GO:0010811 positive regulation of cell-substrate adhesion(38)
GO:0010332 response to gamma radiation(38)
GO:0045429 positive regulation of nitric oxide biosynthetic process(35)
GO:0030593 neutrophil chemotaxis(34)
GO:0007159 leukocyte cell-cell adhesion(33)
GO:0006953 acute-phase response(33)
GO:0046677 response to antibiotic(32)
GO:0006654 phosphatidic acid biosynthetic process(32)
GO:0006956 complement activation(31)
GO:0044344 cellular response to fibroblast growth factor stimulus(30)
GO:0050830 defense response to Gram-positive bacterium(27)
GO:0014823 response to activity(27)
GO:0045599 negative regulation of fat cell differentiation(26)
GO:0010043 response to zinc ion(24)
GO:0043200 response to amino acid stimulus(23)
GO:0090023 positive regulation of neutrophil chemotaxis(21)
GO:0050715 positive regulation of cytokine secretion(21)
GO:0030449 regulation of complement activation(21)
GO:0019915 lipid storage(19)
GO:0048246 macrophage chemotaxis(17)
GO:0010243 response to organonitrogen compound(16)
GO:0006646 phosphatidylethanolamine biosynthetic process(16)
GO:0050930 induction of positive chemotaxis(15)
GO:0050829 defense response to Gram-negative bacterium(15)
GO:0055093 response to hyperoxia(14)
GO:0050995 negative regulation of lipid catabolic process(13)
GO:0042340 keratan sulfate catabolic process(13)
GO:0030194 positive regulation of blood coagulation(13)
GO:0051602 response to electrical stimulus(12)
GO:0042832 defense response to protozoan(12)
GO:0030851 granulocyte differentiation(12)
GO:0014912 negative regulation of smooth muscle cell migration(12)
GO:0006957 complement activation, alternative pathway(12)
GO:0002544 chronic inflammatory response(12)
GO:0002237 response to molecule of bacterial origin(12)
GO:1902042 negative regulation of extrinsic apoptotic signaling pathway via death domain receptors(11)
GO:0050708 regulation of protein secretion(11)
GO:0048545 response to steroid hormone stimulus(11)
GO:0045630 positive regulation of T-helper 2 cell differentiation(11)
GO:0010269 response to selenium ion(11)
GO:2000353 positive regulation of endothelial cell apoptotic process(10)
GO:0050832 defense response to fungus(10)
GO:0035634 response to stilbenoid(10)
GO:0034383 low-density lipoprotein particle clearance(10)
GO:0033280 response to vitamin D(10)
GO:0019430 removal of superoxide radicals(10)
Down
Non-dir.
Up
Down
Non-dir.
Up
0
+15
-log(P-value)

Figure S3

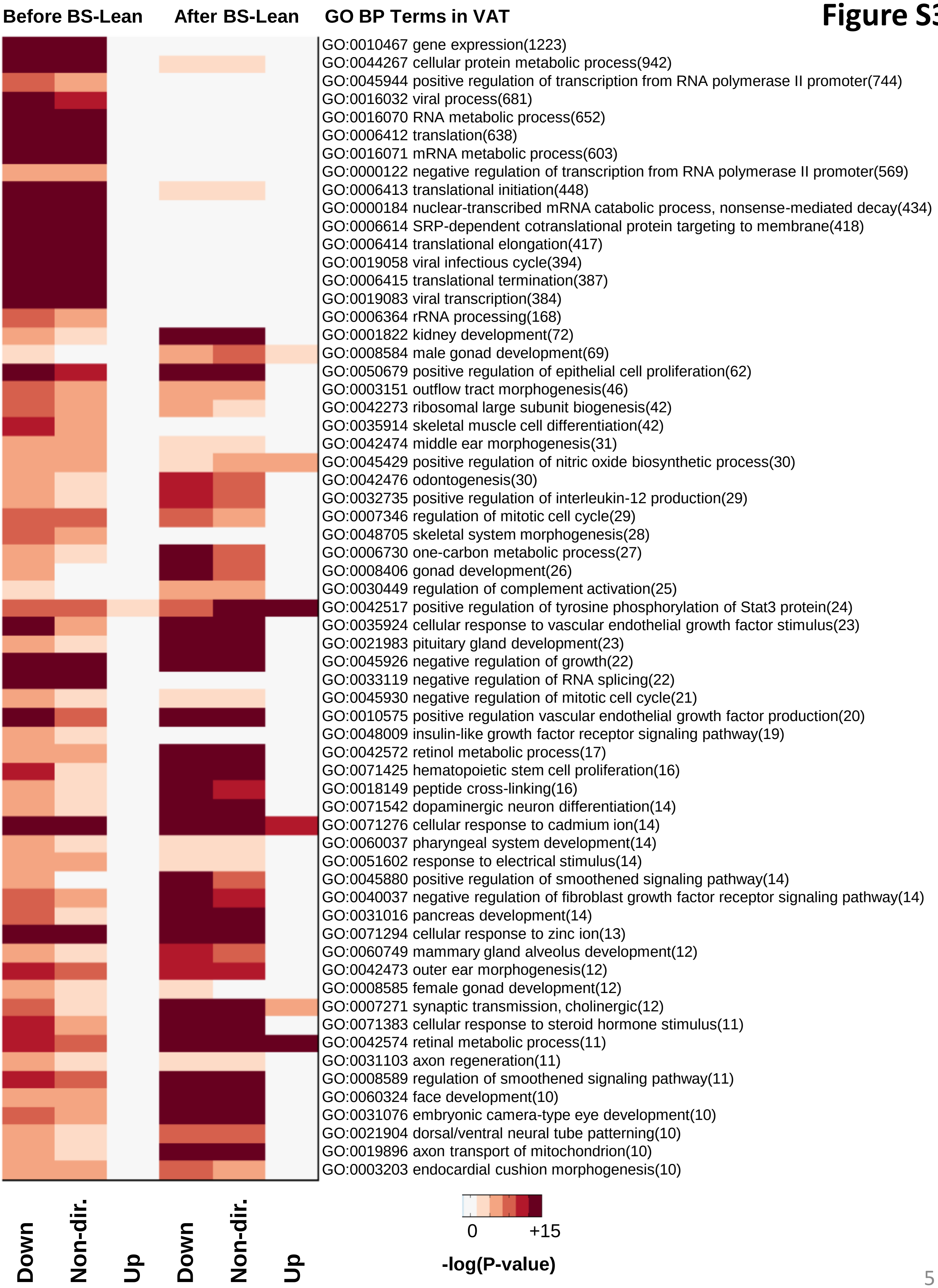

Before BS-Lean
After BS-Lean
GO BP Terms in VAT
GO:0010467 gene expression(1223)
GO:0044267 cellular protein metabolic process(942)
GO:0045944 positive regulation of transcription from RNA polymerase II promoter(744)
GO:0016032 viral process(681)
GO:0016070 RNA metabolic process(652)
GO:0006412 translation(638)
GO:0016071 mRNA metabolic process(603)
GO:0000122 negative regulation of transcription from RNA polymerase II promoter(569)
GO:0006413 translational initiation(448)
GO:0000184 nuclear-transcribed mRNA catabolic process, nonsense-mediated decay(434)
GO:0006614 SRP-dependent cotranslational protein targeting to membrane(418)
GO:0006414 translational elongation(417)
GO:0019058 viral infectious cycle(394)
GO:0006415 translational termination(387)
GO:0019083 viral transcription(384)
GO:0006364 rRNA processing(168)
GO:0001822 kidney development(72)
GO:0008584 male gonad development(69)
GO:0050679 positive regulation of epithelial cell proliferation(62)
GO:0003151 outflow tract morphogenesis(46)
GO:0042273 ribosomal large subunit biogenesis(42)
GO:0035914 skeletal muscle cell differentiation(42)
GO:0042474 middle ear morphogenesis(31)
GO:0045429 positive regulation of nitric oxide biosynthetic process(30)
GO:0042476 odontogenesis(30)
GO:0032735 positive regulation of interleukin-12 production(29)
GO:0007346 regulation of mitotic cell cycle(29)
GO:0048705 skeletal system morphogenesis(28)
GO:0006730 one-carbon metabolic process(27)
GO:0008406 gonad development(26)
GO:0030449 regulation of complement activation(25)
GO:0042517 positive regulation of tyrosine phosphorylation of Stat3 protein(24)
GO:0035924 cellular response to vascular endothelial growth factor stimulus(23)
GO:0021983 pituitary gland development(23)
GO:0045926 negative regulation of growth(22)
GO:0033119 negative regulation of RNA splicing(22)
GO:0045930 negative regulation of mitotic cell cycle(21)
GO:0010575 positive regulation vascular endothelial growth factor production(20)
GO:0048009 insulin-like growth factor receptor signaling pathway(19)
GO:0042572 retinol metabolic process(17)
GO:0071425 hematopoietic stem cell proliferation(16)
GO:0018149 peptide cross-linking(16)
GO:0071542 dopaminergic neuron differentiation(14)
GO:0071276 cellular response to cadmium ion(14)
GO:0060037 pharyngeal system development(14)
GO:0051602 response to electrical stimulus(14)
GO:0045880 positive regulation of smoothened signaling pathway(14)
GO:0040037 negative regulation of fibroblast growth factor receptor signaling pathway(14)
GO:0031016 pancreas development(14)
GO:0071294 cellular response to zinc ion(13)
GO:0060749 mammary gland alveolus development(12)
GO:0042473 outer ear morphogenesis(12)
GO:0008585 female gonad development(12)
GO:0007271 synaptic transmission, cholinergic(12)
GO:0071383 cellular response to steroid hormone stimulus(11)
GO:0042574 retinal metabolic process(11)
GO:0031103 axon regeneration(11)
GO:0008589 regulation of smoothened signaling pathway(11)
GO:0060324 face development(10)
GO:0031076 embryonic camera-type eye development(10)
GO:0021904 dorsal/ventral neural tube patterning(10)
GO:0019896 axon transport of mitochondrion(10)
GO:0003203 endocardial cushion morphogenesis(10)
Down
Non-dir.
Up
Down
Non-dir.
Up
0
+15
-log(P-value)

Figure S4

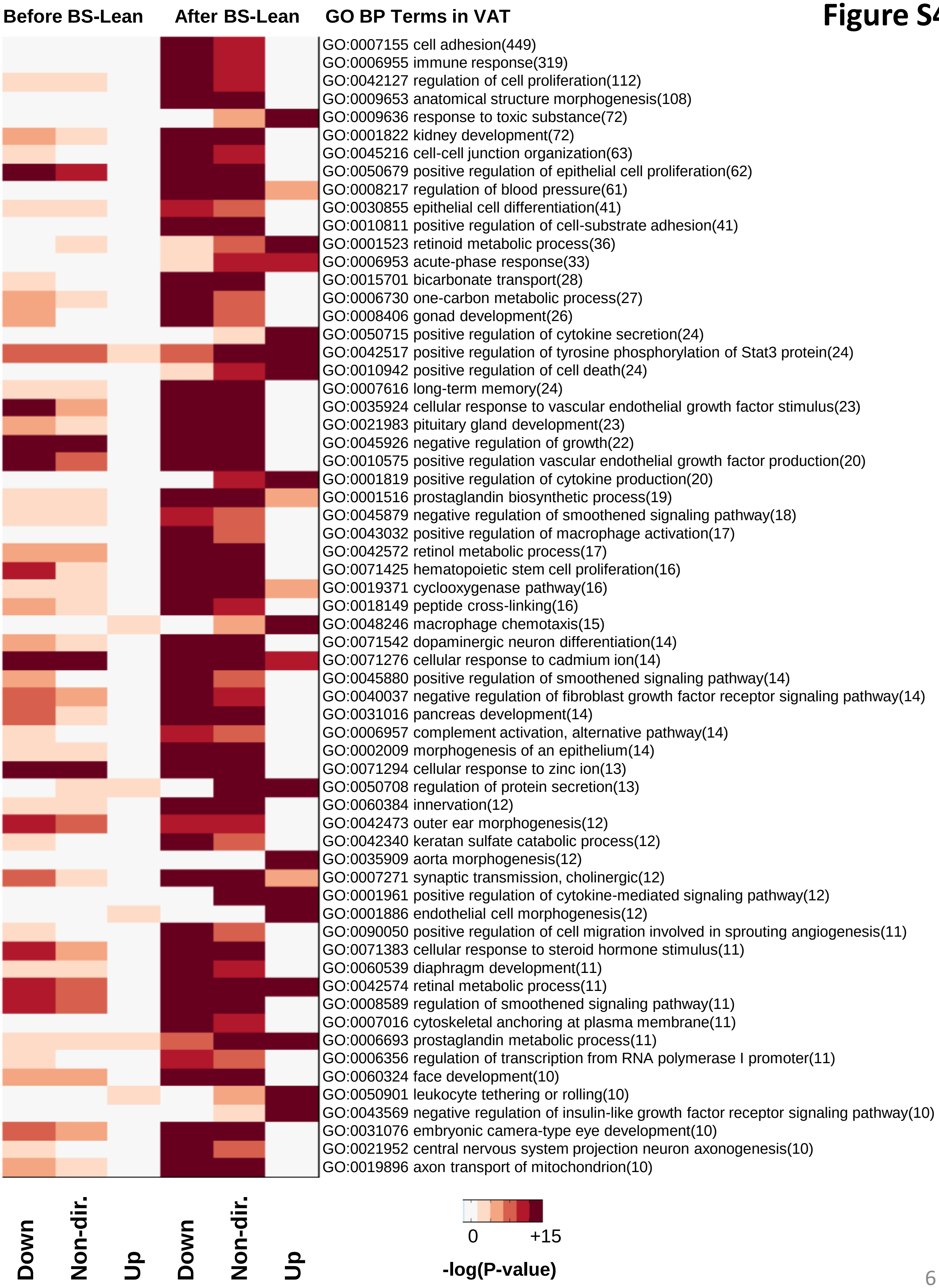

Before BS-Lean
After BS-Lean
GO BP Terms in VAT
GO:0007155 cell adhesion(449)
GO:0006955 immune response(319)
GO:0042127 regulation of cell proliferation(112)
GO:0009653 anatomical structure morphogenesis(108)
GO:0009636 response to toxic substance(72)
GO:0001822 kidney development(72)
GO:0045216 cell-cell junction organization(63)
GO:0050679 positive regulation of epithelial cell proliferation(62)
GO:0008217 regulation of blood pressure(61)
GO:0030855 epithelial cell differentiation(41)
GO:0010811 positive regulation of cell-substrate adhesion(41)
GO:0001523 retinoid metabolic process(36)
GO:0006953 acute-phase response(33)
GO:0015701 bicarbonate transport(28)
GO:0006730 one-carbon metabolic process(27)
GO:0008406 gonad development(26)
GO:0050715 positive regulation of cytokine secretion(24)
GO:0042517 positive regulation of tyrosine phosphorylation of Stat3 protein(24)
GO:0010942 positive regulation of cell death(24)
GO:0007616 long-term memory(24)
GO:0035924 cellular response to vascular endothelial growth factor stimulus(23)
GO:0021983 pituitary gland development(23)
GO:0045926 negative regulation of growth(22)
GO:0010575 positive regulation vascular endothelial growth factor production(20)
GO:0001819 positive regulation of cytokine production(20)
GO:0001516 prostaglandin biosynthetic process(19)
GO:0045879 negative regulation of smoothened signaling pathway(18)
GO:0043032 positive regulation of macrophage activation(17)
GO:0042572 retinol metabolic process(17)
GO:0071425 hematopoietic stem cell proliferation(16)
GO:0019371 cyclooxygenase pathway(16)
GO:0018149 peptide cross-linking(16)
GO:0048246 macrophage chemotaxis(15)
GO:0071542 dopaminergic neuron differentiation(14)
GO:0071276 cellular response to cadmium ion(14)
GO:0045880 positive regulation of smoothened signaling pathway(14)
GO:0040037 negative regulation of fibroblast growth factor receptor signaling pathway(14)
GO:0031016 pancreas development(14)
GO:0006957 complement activation, alternative pathway(14)
GO:0002009 morphogenesis of an epithelium(14)
GO:0071294 cellular response to zinc ion(13)
GO:0050708 regulation of protein secretion(13)
GO:0060384 innervation(12)
GO:0042473 outer ear morphogenesis(12)
GO:0042340 keratan sulfate catabolic process(12)
GO:0035909 aorta morphogenesis(12)
GO:0007271 synaptic transmission, cholinergic(12)
GO:0001961 positive regulation of cytokine-mediated signaling pathway(12)
GO:0001886 endothelial cell morphogenesis(12)
GO:0090050 positive regulation of cell migration involved in sprouting angiogenesis(11)
GO:0071383 cellular response to steroid hormone stimulus(11)
GO:0060539 diaphragm development(11)
GO:0042574 retinal metabolic process(11)
GO:0008589 regulation of smoothened signaling pathway(11)
GO:0007016 cytoskeletal anchoring at plasma membrane(11)
GO:0006693 prostaglandin metabolic process(11)
GO:0006356 regulation of transcription from RNA polymerase I promoter(11)
GO:0060324 face development(10)
GO:0050901 leukocyte tethering or rolling(10)
GO:0043569 negative regulation of insulin-like growth factor receptor signaling pathway(10)
GO:0031076 embryonic camera-type eye development(10)
GO:0021952 central nervous system projection neuron axonogenesis(10)
GO:0019896 axon transport of mitochondrion(10)
Down
Non-dir.
Up
Down
Non-dir.
Up
0
+15
-log(P-value)

A. **Visceral Adipose Tissue** **Figure S5**

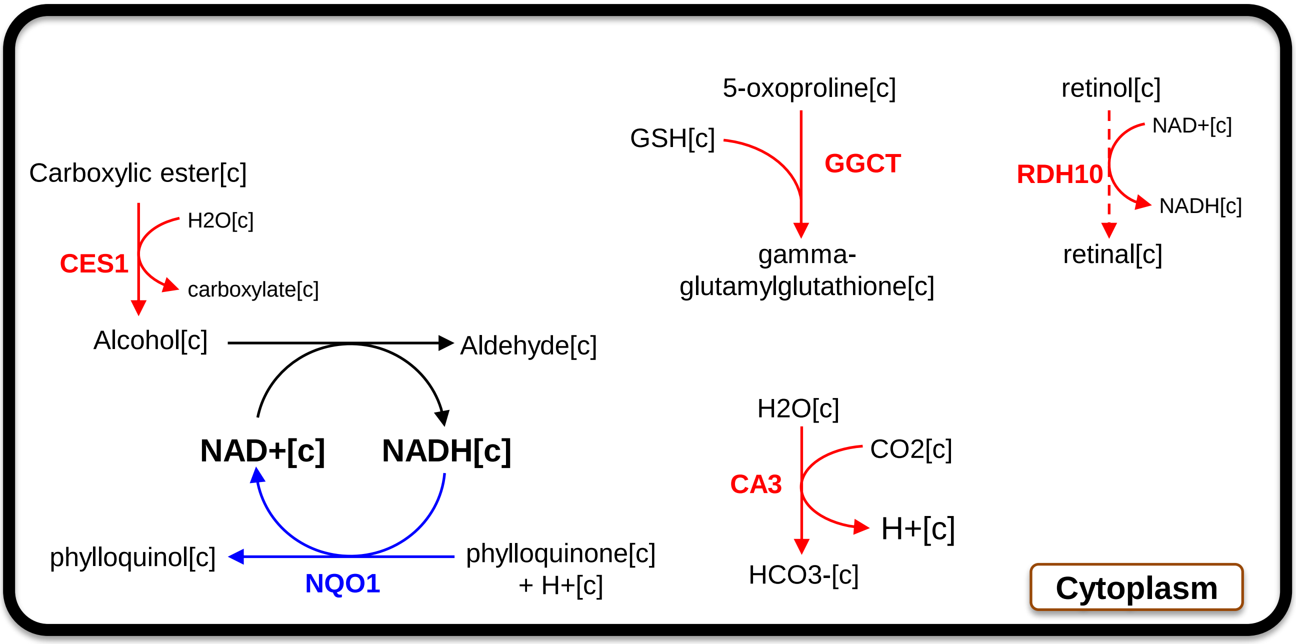

B. **Subcutaneous Adipose Tissue**

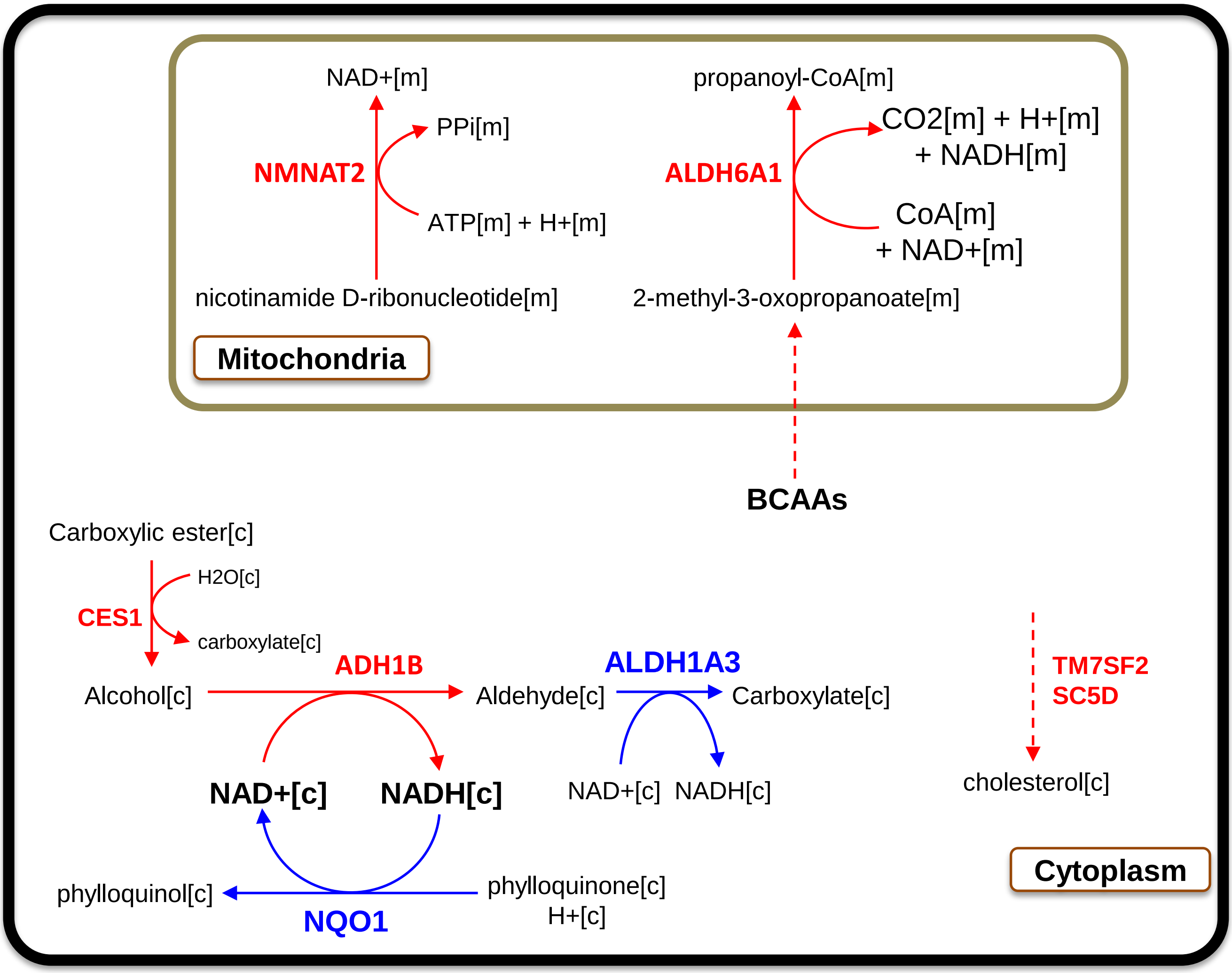

Supplement: Supplementary Information [file srep14841-s1.pdf]
